# Switching PD-based sliding mode control for hovering of a tilting-thruster underwater robot

**DOI:** 10.1371/journal.pone.0194427

**Published:** 2018-03-16

**Authors:** Sangrok Jin, Jeongae Bak, Jongwon Kim, TaeWon Seo, Hwa Soo Kim

**Affiliations:** 1 School of Mechanical Engineering, Pusan National University, Republic of Korea; 2 School of Mechanical and Aerospace Engineering, Seoul National University, Republic of Korea; 3 Department of Mechanical Engineering, Hanyang University, Republic of Korea; 4 Department of Mechanical System Engineering, Kyonggi University, Republic of Korea; Lanzhou University of Technology, CHINA

## Abstract

This paper presents a switching PD-based sliding mode control (PD-SMC) method for the 6-degree-of-freedom (DOF) hovering motion of the underwater robot with tilting thrusters. Four thrusters of robot can be tilted simultaneously in the horizontal and vertical directions, and the 6-DOF motion is achieved by switching between two thruster configurations. Therefore, the tilting speed of thruster becomes the most essential parameter to determine the stability of hovering motion. Even though the previous PD control ensures stable hovering motion within a certain ranges of tilting speed, a PD-SMC is suggested in this paper by combining PD control with sliding mode control in order to achieve acceptable hovering performance even at the much lower tilting speeds. Also, the sign function in the sliding mode control is replaced by a sigmoid function to reduce undesired chattering. Simulations show that while PD control is effective only for tilting duration of 600 ms, the PD-based sliding mode control can guarantee the stable hovering motion of underwater robot even for the tilting duration of up to 1500 ms. Extensive experimental results confirm the hovering performance of the proposed PD-SMC method is much superior to that of PD method for much larger tilting durations.

## Introduction

As the robot technologies have been developed, the missions for underwater robots have rapidly expanded from monitoring or inspection to practical underwater tasks that are similar to those of a human diver. Fernandez *et al*. developed an underwater robot arm for grasping in shallow water for an autonomous underwater vehicle (AUV) [1]. Asokan *et al*. proposed the optimum positioning algorithm of underwater robotic vehicle with a 7-DOF manipulator [2]. Ambar *et al*. developed a dual-arm underwater robot and controlled using the resolved acceleration control method [3]. Many underwater manipulators are designed and controlled under the assumption that the base of the manipulator is sufficiently stationary. Thus, the hovering control that maintains the position and orientation of underwater robots against disturbances is the most important performance characteristic of underwater robotic platforms for successfully completing underwater tasks with manipulators.

**Fig 1:**
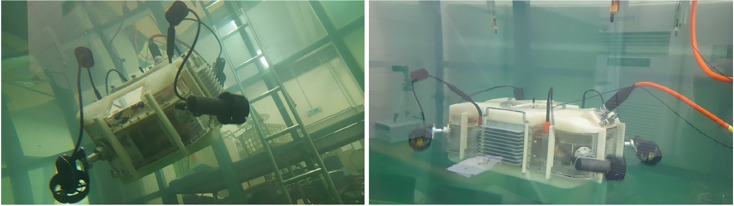
Tilted thrusting underwater robot (TTURT).

**Fig 2:**
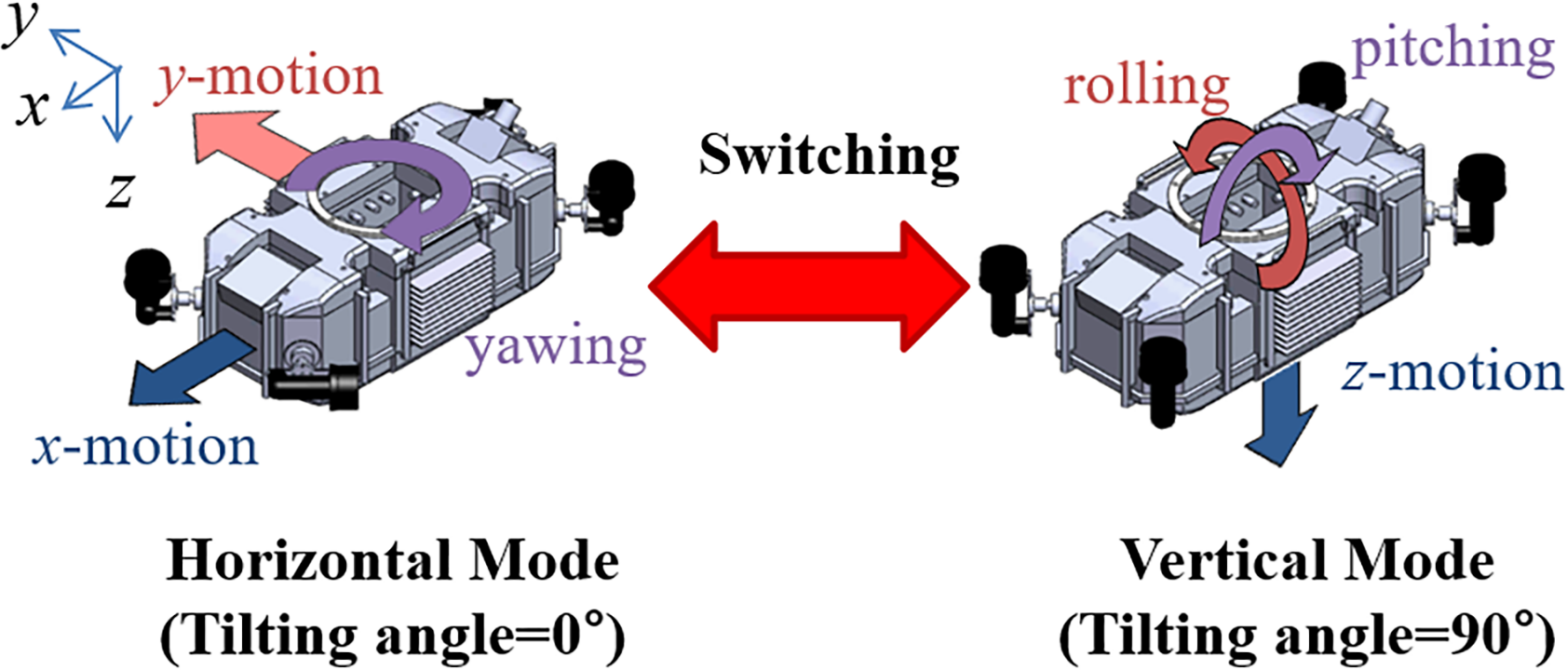
Switching control between two subsystems according to thruster configuration.

We previously developed a tilting-thruster underwater robot (TTURT) that achieves 6-DOF hovering motion with only four thrusters, as shown in Fig 1. This robot is a remotely operated vehicle (ROV) which has a tether, and it can supply power to the attached manipulator. The two front thrusters are mechanically connected with one servo motor and tilted simultaneously, and the two rear thrusters can be tilted in the same way. Hovering control with a smooth tilting angle is a challenging problem because of the nonlinear thrusting vector [4]. We simplified the control algorithm, and a selective switching PD controller was designed for 6-DOF hovering control [5]. This controller was operated only in two tilting angles: 0° for the horizontal configuration, and 90° for the vertical configuration, as shown in Fig 2.

The 6-DOF system is divided into two 3-DOF subsystems. While the horizontal mode can control *x*- and *y*-directional linear motions and yawing, the vertical mode can control *z*-directional linear motion, rolling, and pitching. The selective switching logic chooses one of two subsystems which has a large motion error. Then, the thrusters are tilted into the relevant configuration, and the chosen subsystem is controlled by the PD controller. These switching process are repeated in real time. The switching control algorithm is very sensitive to the tilting speed of thruster. As the tilting speed becomes faster, the better control performance can be obtained theoretically. However, the tilted thrusting mechanism has a critical limitation of the tilting speed. We cannot increase the tilting speed infinitely because of the physical limitations such as torque limit, motor size, and wear of the rotary seal. For the PD controller in previous study [5], its performance can be guaranteed only when the tilting duration is less than 600 ms. It is very tough condition on compact design with tilting mechanism, and too fast tilting motion can generate undesired reaction torque [6]. From this viewpoint, the advanced control methodology seems indispensable for obtaining the robust performance against slower tilting speed.

Sliding mode control is well known as one of the robust control algorithms, and it is being wide range of applications with combination of various control algorithms. Fei and Jhou proposed an adaptive sliding model control with fuzzy compensator for MEMS triaxial gyroscope [7]. Fei *et al*. presented adaptive sliding model control strategies using radial basis function neural network [8] and double loop recurrent neural network [9]. Sliding mode control has been also widely adopted positioning of underwater robots due to its robustness. Chatchanayuenyong and Parnichkun proposed a neural-network based-time optimal sliding mode control method for an autonomous underwater robot [10]. Healey and Lienard designed a multivariable sliding mode control for autonomous diving and steering of unmanned underwater vehicle. [11]. Note that a sliding mode controller is a model-based control method. However, by combining it with PD or PID control tactfully, the complicated model-based terms can be removed to derive a model-free controller. Parra-vega *et al*. proposed a dynamic sliding PID controller for the tracking of robot manipulators [12]. Peng and Chen presented an integrated PID-based sliding mode state estimation and control for piezoelectric actuators [13]. Ouyang *et al*. designed a PD controller with sliding mode control for trajectory tracking of a robotic system [14].

This paper applies a PD-based sliding mode control (PD-SMC) to each subsystem for switching control of TTURT. The proposed PD-SMC is designed by combining the sliding mode control with the PD control, which can lead to more acceptable performance even for much larger tilting duration in comparison with the previous PD control. In order to handle undesired chattering of sliding mode control, the sign function in the sliding mode term is replaced with a sigmoid function. Also, the control gains of proposed PD-SMC are optimally chosen in such a manner to minimize the steady-state error within a 10% overshoot. The stability of proposed PD-SMC is verified by using Lyapunov function analysis. Extensive simulations and experiments are carried out, which demonstrate the proposed PD-SMC shows the superiority to the previous PD even though the tilting duration is significantly increased.

The contributions of this paper are summarized as follow: first, a switching PD-based sliding mode control is successfully designed for tilting thrust mechanism of TTURT. As a result, more robust control performance can be achieved during experiments for tilting duration varying from 300 ms to 1500 ms compared to the PD control. Second, the sigmoid function in PD-SMC has shown to improve performance of the tilting thruster mechanism. Third, the stability of PD-SMC is theoretically verified via a Lyapunov function analysis and the stability of switching control for PD-SMC can be ensured experimentally even for various tilting durations by restricting the error within a certain range strictly.

## Dynamic model for proposed TTURT

TTURT was deliberately designed to simplify the dynamic model, which can be derived based on some assumptions. The robot body has a three planes symmetric shape and neutral buoyancy. The center of mass and center of buoyancy coincide with the origin of the body-fixed frame. The angular momentum of the rotating thruster propeller is negligible because of the relatively small inertia. These conditions make many terms in the dynamic model disappear. The overall size of TTURT is 326 mm (W) × 755 mm (L) × 280 mm (H), and its weight is 57.1 kg. More detailed specifications are described in our previous paper [5].

The switching control algorithm divides the 6-DOF model into two 3-DOF sub-models according to the thruster configurations. The sub-model of the horizontal configuration is used for a tilting angle of 0° and involves the *x-* and *y*-directional linear motions and the yawing rotational motion. The model of the vertical configuration is used for a tilting angle of 90° and involves the *z*-directional linear motion and the rolling and pitching rotational motions. The subsystem of the horizontal mode is independent, but the subsystem of vertical mode can affect the *x-* and *y*-directional motions similar to quadrotors. For example, *z*-directional motion with a pitch angle in the body-fixed frame leads to *x*-directional motion in the earth-fixed frame. In general, since the hovering motion has small displacement, the side effect of the vertical-mode motion can be sufficiently compensated by switching to horizontal mode. We assumed that the *x-* and *y-*directional motions are restricted in the vertical mode.

The velocities of each subsystem in the body-fixed frame are *v*_*hor*_ = [*u*, *v*, 0, 0, 0, *r*]^T^ and *v*_*ver*_ = [0, 0, *w*, *p*, *q*, 0]^T^. *u*, *v*, and *w* are the linear velocities in the *x*, *y*, and *z* directions, while *p*, *q*, and *r* are the roll, pitch, and yaw angular velocities, respectively. The position and orientation of each subsystem in the earth-fixed frame are *η*_*hor*_ = [*x*, *y*, 0, 0, 0, *ψ*]^T^ and *η*_*ver*_ = [0, 0, *z*, *φ*, *θ*, 0]^T^. *x*, *y*, and *z* describe the position, while *φ*, *θ*, and *ψ* describe the orientation. The body-fixed velocity and earth-fixed displacement are related by the transformation matrix **J** as follow [15]:

$$v_{hor}=J^{-1}(v_{hor}){\dot{\eta }}_{hor},$$ (1)

$$v_{ver}=J^{-1}(v_{ver}){\dot{\eta }}_{ver}.$$ (2)

### Body-fixed motion equations

The motion equations of the robot in the body-fixed frame are derived from Newton-Euler formulation as follow [13]:

$$M{\dot{v}}_{hor}+C(v_{hor})v_{hor}+D(v_{hor})v_{hor}=B(0{}^{\circ})f,$$ (3)

$$M{\dot{v}}_{ver}+C(v_{ver})v_{ver}+D(v_{ver})v_{ver}=B(90{}^{\circ})f.$$ (4)

The subscripts indicate horizontal or vertical mode. **M** and **C** are the inertia matrix and centripetal matrix with added mass, respectively. **D** is the hydrodynamic damping matrix. **M** and **D** are diagonal and positive definite matrices, and **C** is a skew-symmetric matrix. The thrust force vector from the four thrusters is *f* = [*f*_1_, *f*_2_, *f*_3_, *f*_4_]^T^. The details of the dynamics terms are described in the appendix.

Note that the TTURT’s thrusters are tilted at four vertices of the robot body so that the thrust vectoring matrix **B** has nonlinear terms with a sinusoidal function for the tilting angles [4]. Transient time of thrust force is very short relative to tilting duration between horizontal and vertical mode. Tilting angle is the dominant factor of thrust vector, and matrix **B** is time-invariant. In this switching control, since the all thrusters are synchronously tilted, the thrust vectoring matrix is simply given by

$$B(\alpha )=\left[\begin{array}{cccc} \frac{\cos\alpha }{\sqrt{2}} & \frac{\cos\alpha }{\sqrt{2}} & \frac{\cos\alpha }{\sqrt{2}} & \frac{\cos\alpha }{\sqrt{2}}\\ \frac{\cos\alpha }{\sqrt{2}} & -\frac{\cos\alpha }{\sqrt{2}} & \frac{\cos\alpha }{\sqrt{2}} & -\frac{\cos\alpha }{\sqrt{2}}\\ -\sin\alpha & -\sin\alpha & -\sin\alpha & -\sin\alpha \\ \frac{W\sin\alpha }{2} & -\frac{W\sin\alpha }{2} & -\frac{W\sin\alpha }{2} & \frac{W\sin\alpha }{2}\\ \frac{L\sin\alpha }{2} & \frac{L\sin\alpha }{2} & -\frac{L\sin\alpha }{2} & -\frac{L\sin\alpha }{2}\\ \frac{\left(W+L\right)\cos\alpha }{2\sqrt{2}} & -\frac{\left(W+L\right)\cos\alpha }{2\sqrt{2}} & -\frac{\left(W+L\right)\cos\alpha }{2\sqrt{2}} & \frac{\left(W+L\right)\cos\alpha }{2\sqrt{2}} \end{array}\right],$$ (5)

where *α*, *W* and *L* are tilting angle of thrusters, width and length of the robot body, respectively.

### Earth-fixed motion equations

Hovering control for an underwater task is used to maintain the position and orientation of the robot relative to an externally fixed task object. The motion equation in the body-fixed frame, (3) and (4), can be transformed into the earth-fixed motion equation using the relations (1) and (2) as follows:

$$M_{\eta }(\eta )\ddot{\eta }+C_{\eta }(\eta ,v)\dot{\eta }+D_{\eta }(\eta ,v)\dot{\eta }=J^{-T}Bf.$$ (6)

where

$$\begin{array}{l} M_{\eta }=J^{-T}MJ^{-1},\\ C_{\eta }=J^{-T}\left[C-MJ^{-1}\dot{J}\right]J^{-1},\\ D_{\eta }=J^{-T}DJ^{-1}. \end{array}$$ (7)

The subscripts for the thruster configuration are omitted because both motion equations are transformed in the same way. The earth-fixed motion equations satisfy the following properties [16]:

$$1)\;M_{\eta }(\eta )=M_{\eta }^{T}(\eta )>0,\forall \eta .$$ (8)

$$2)\;q^{T}[{\dot{M}}_{\eta }(\eta )-2C_{\eta }(\eta ,v)]q=0,\forall q,\eta ,v.$$ (9)

$$3)\;D_{\eta }(\eta ,\;v)>0,\forall \eta ,v.$$ (10)

## Switching PD-SMC design

**Fig 3:**
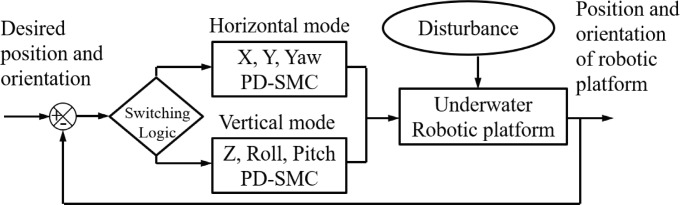
Block diagram of switching PD-SMC.

The switching control is one of the solutions for resolving the complicated nonlinear problem of the tilting thrust mechanism. The 6-DOF motion is controlled by switching between two PD-SMCs. Each PD-SMC deals with 3-DOF motion according to the thruster configuration. A block diagram of the method is shown in Fig 3. The switching logic is as follows:

1. The position and orientation errors are non-dimensionalized with reference values. The position reference is 0.1 m, and the orientation reference is 10°.
2. Dimensionless errors are divided into two groups depending on the thruster configuration.
3. The maximum errors of each group are compared, and the sub-controller with the larger maximum error is chosen.
4. Thrusters are tilted into the selected configuration, and the PD-SMC is applied.
5. These processes are repeated over the control period.

                Swtiching Logic

Ref_pos = 0.1; Ref_ori = 10;

while(control)

    MaxError_hor = max(Err_x/Ref_pos,         Err_y/Ref_pos, Err_yaw/Ref_ori);

    MaxError_ver = max(Err_z/Ref_pos, Err_roll/Ref_ori, Err_pitch/Ref_ori);

        if MaxError_hor> = MaxError_ver

            TiltAngle = 0;

            Horizontal mode Control(Err_x, Err_y, Err_yaw);

        else

            TiltAngle = 90;

            Vertical mode Control(Err_z, Err_roll, Err_pitch);

        end

end

The thrusters are temporarily shut down during the tilting to prevent undesired reaction forces and moments. Note that the system is fully uncontrollable when the thrusters are tilting so that the tilting speed may have a crucial influence on the performance of the switching control.

### PD-based sliding mode control law

The design of the sliding mode control begins with the definition of a sliding surface. The error vector *e* and its derivative are defined as follow:

$$e=\eta_{d}-\eta ,$$ (11)

$$\dot{e}=\dot{\eta }=-Jv,$$ (12)

where *η*_*d*_ is the constant desired position and orientation in the earth-fixed frame. A sliding surface is selected as follows:

$$s=\dot{e}+\Lambda e,$$ (13)

where **Λ** is a diagonal positive matrix whose components are the sliding surface slope constants.

Conventional sliding mode control for a marine vehicle consists of model-based terms and a sliding mode term [17]. The model-based terms are apt to be affected by modeling errors. To overcome this defect of sliding mode control, the model-free PD-SMC was designed as follows:

$$f=B^{+}J^{T}[K_{P}e+K_{D}\dot{e}+P\mathrm{sign}(s)],$$ (14)

where **sign**(*s*) = [*sign*(*s*_1_)⋯*sign*(*s*_3_)]^*T*^ and *s*_*i*_ is *i-*th element of vector *s*. **K**_*P*_ and **K**_*D*_ are the proportional and derivative control gains of the PD control, **P** is the sliding mode control gain, and the superscript ‘+’ means the Moore-Penrose pseudoinverse. **K**_*P*_, **K**_*D*_, and **P** are positive and diagonal.

### Sigmoid function for avoidance of chattering

The sliding model control often suffers from chattering, which results in high-frequency oscillations in the control input and performance deterioration. The discontinuous sign function of the sliding model term is a major cause. Many studies have tried to solve this problem. In general cases, a saturation function is substituted for the sign function [14]. Lee and Utkin reviewed various attempts to reduce chattering [18]. Fallaha *et al*. reduced the chattering in the control input by using an exponential reaching law [19]. Kim *et al*. replaced the sign function with a sigmoid function to reduce chattering [20].

**Fig 4:**
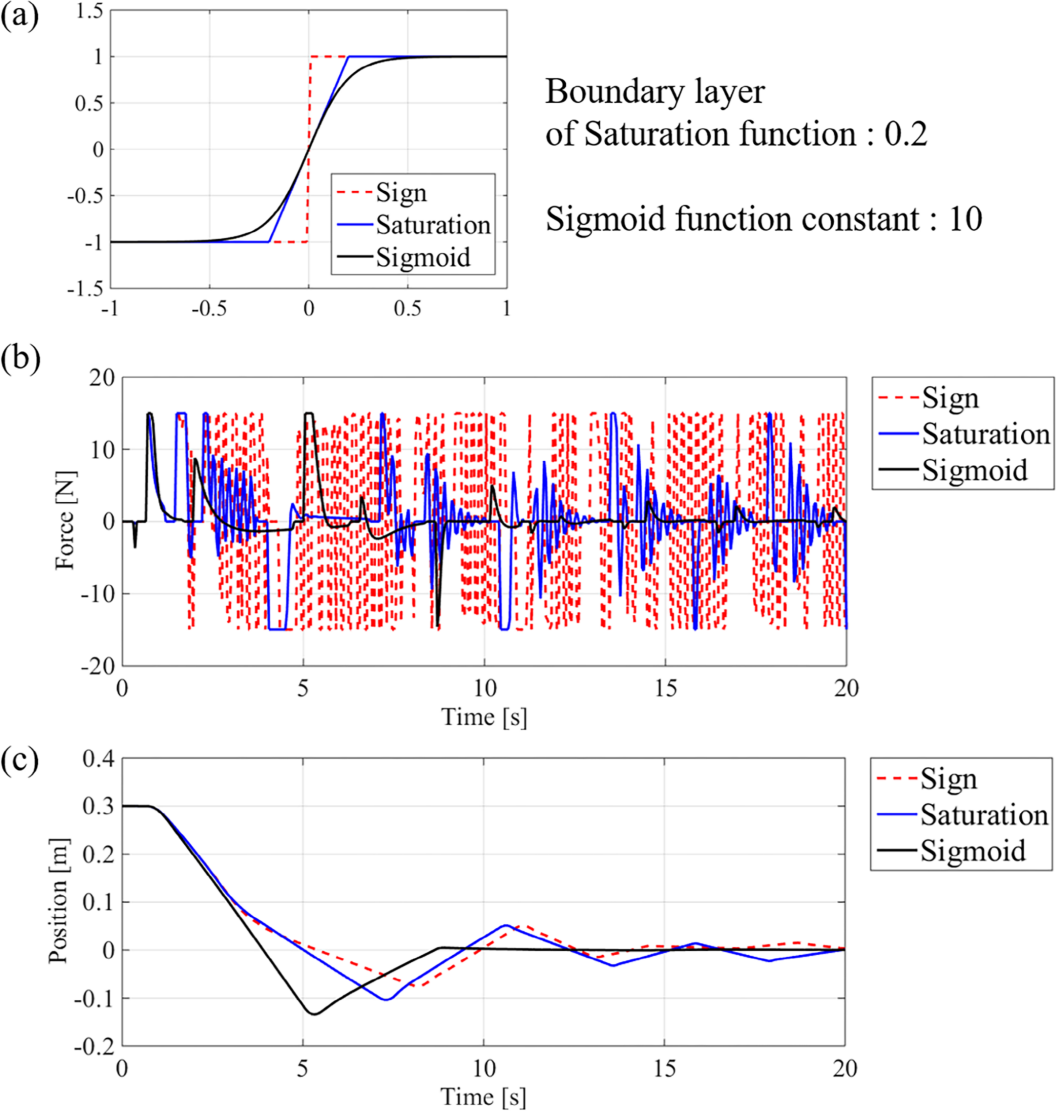
Comparison with sign, saturation, and sigmoid function. (a) plots of each function, (b) control input of thruster 1 in simulations, (c) x-directional motion in simulations.

We replace the sign function in (14) with the following sigmoid function to reduce chattering:

$$sigmoid(s,a)=\frac{2}{1+e^{-as}}-1,$$ (15)

where *a* is a positive constant. The plots of sign, saturation, and sigmoid functions are compared in Fig 4(A). The advantages of sigmoid the function are continuity as well as smoothness. The control input to the thrust force is too large and oscillates while using the sign and saturation functions, as shown in Fig 4(B). It is noted that the thrusters have a little delay when generating the desired force due to the motor dynamics and hydrodynamics [6]. Since the actual system cannot follow the control input with the sign and saturation functions, chattering inevitably occurs. The sigmoid function generates much smoother control input as shown in Fig 4(B) so that the control performance of sigmoid function becomes better than those of other functions, as shown in Fig 4(C).

### Stability analysis

The stability of each PD-SMC is proven based on Lyapunov stability theory. Consider the following Lyapunov function candidate *V*:

$$V=\frac{1}{2}{\left(\begin{array}{c} \dot{e}\\ e \end{array}\right)}^{T}\left[\begin{array}{cc} M_{\eta } & 0\\ 2\Lambda M_{\eta } & K_{P}+\Lambda K_{D} \end{array}\right]\left(\begin{array}{c} \dot{e}\\ e \end{array}\right).$$ (16)

It is noted that the matrix of Lyapunov function candidate *V* is the lower triangular block matrix whose eigenvalues are determined by its diagonal matrices. Since **M**_*η*_, **K**_*P*_, **Λ** and **K**_*D*_ are all positive definite, the Lyapunov function candidate *V* is obviously positive. By using properties in (8)–(10) and the control law in (14), the derivative of *V* can be derived as follows:

$$\begin{array}{l} \dot{V}={\dot{e}}^{T}M_{\eta }\ddot{e}+{\dot{e}}^{T}\Lambda M_{\eta }\dot{e}+e^{T}\Lambda M_{\eta }\ddot{e}+{\dot{e}}^{T}(K_{P}+\Lambda K_{D})e+\frac{1}{2}\left({\dot{e}}^{T}{\dot{M}}_{\eta }\dot{e}+2e^{T}\Lambda {\dot{M}}_{\eta }\dot{e}\right)\\ \;={\dot{e}}^{T}(-C_{\eta }\dot{e}-D_{\eta }\dot{e}-\tau_{\eta })+{\dot{e}}^{T}\Lambda M_{\eta }\dot{e}+e^{T}\Lambda (-C_{\eta }\dot{e}-D_{\eta }\dot{e}-\tau_{\eta })\\ \;+{\dot{e}}^{T}K_{P}e+e^{T}\Lambda K_{D}\dot{e}+\frac{1}{2}\left({\dot{e}}^{T}{\dot{M}}_{\eta }\dot{e}+e^{T}\Lambda {\dot{M}}_{\eta }\dot{e}+{\dot{e}}^{T}\Lambda {\dot{M}}_{\eta }e\right)\\ \;=-\frac{1}{2}{\dot{e}}^{T}\left[{\dot{M}}_{\eta }-2C_{\eta }\right]\dot{e}-{\dot{e}}^{T}D_{\eta }\dot{e}-{\dot{e}}^{T}K_{D}\dot{e}-{\dot{e}}^{T}P\mathrm{sigmoid}(s,a)\\ \;+{\dot{e}}^{T}\Lambda M_{\eta }\dot{e}-e^{T}\Lambda C_{\eta }\dot{e}-e^{T}\Lambda D_{\eta }\dot{e}-e^{T}\Lambda K_{P}e-e^{T}\Lambda P\mathrm{sigmoid}(s,a)\\ \;+\frac{1}{2}\left(e^{T}\Lambda {\dot{M}}_{\eta }\dot{e}+{\dot{e}}^{T}\Lambda {\dot{M}}_{\eta }e\right)\\ \;=-{\dot{e}}^{T}D_{\eta }\dot{e}-{\dot{e}}^{T}{Κ}_{D}\dot{e}-{\dot{e}}^{T}P\mathrm{sigmoid}(s,a)+{\dot{e}}^{T}\Lambda M_{\eta }\dot{e}\\ \;-e^{T}\Lambda C_{\eta }\dot{e}-e^{T}\Lambda D_{\eta }\dot{e}-e^{T}\Lambda K_{p}e-e^{T}\Lambda P\mathrm{sigmoid}(s,a)+{\dot{e}}^{T}\Lambda {\dot{M}}_{\eta }e\\ \;=-{\left(\begin{array}{c} \dot{e}\\ e \end{array}\right)}^{T}Q\left(\begin{array}{c} \dot{e}\\ e \end{array}\right)-s^{T}P\mathrm{sigmoid}(s,a) \end{array}$$ (17)

where,

$$Q=\left[\begin{array}{cc} D_{\eta }+K_{D}-\Lambda M_{\eta } & 0\\ \Lambda (C_{\eta }+D_{\eta }-\Lambda {\dot{M}}_{\eta }) & \Lambda K_{P} \end{array}\right],\;\mathrm{sigmoid}(s,a)={\left[sigmoid(s_{1},a_{1})\cdots sigmoid(s_{3},a_{3})\right]}^{T}$$ (18)

Recall that **Q** is positive definite if its minimum eigenvalue is positive and that the eigenvalues of **Q** are equal to the diagonal elements since **Q** is a lower triangular block matrix. **M**_*η*_, **D**_*η*_, **K**_*P*_, **K**_*D*_, and **Λ** are all positive definite. Therefore, it can be guaranteed that **Q** is positive definite for a suitable sliding surface slope constant matrix **Λ**. If **P** is also positive definite, the derivative in (17) is always negative and, as a result, the PD-SMC is asymptotically stable, which implies that the corresponding error *e* still remains within a certain bound for a finite time 0<*t*<*t*_*finite*_<∞ [21]. Therefore, since the PD-SMC for each control mode ensures the asymptotic stability, the stability of overall switching system can be guaranteed if the maximum period of switching between each control mode is less than *t*_*finite*_. Recall that the minimum period of switching comes from subtracting the tilting duration from the switching period. Appropriate tilting duration is manually set in this paper so that the stability margin can be effectively secured [22].

## Simulations

While the stability of PD-SMC can be proven theoretically, it is difficult to verify the stability of the switching control with PD-SMC because the time required for switching to another sub-controller may vary depending on the situations. Therefore, this time becomes a major factor to determine the performance of the switching control for the TTURT. This system is also fully uncontrollable when the thrusters are tilted since they are turned off to avoid undesired reactions so that the tilting speed plays an important role in the tilting thruster mechanism controlled by switching control. The performance of the switching PD-SMC was therefore simulated with various tilting speeds. The switching PD-SMC was compared with a switching PD control method in order to verify its robustness. Note that since the tilting speed is the combination of the angular velocity and the acceleration/deceleration of the tilting motor, the revolutions per minute (RPM) does not seem suitable for expressing the tilting speed of an actual system. The tilting duration is instead adopted to describe the tilting speed of thruster, which corresponds to the time required for the thruster to change its configuration from one to the other completely.

### Gain optimization

The optimization for control gains of proposed PD-SMC was carried out to ensure the robust performance of the proposed controller through the simulations. Each 3-DOF PD-SMC was optimized separately. The objective function is to minimize a steady-state error under 10% overshoot and positive definite **Q**. The proportional gain **K**_*P*_, derivative gain **K**_*D*_, sliding mode gain **P**, sliding surface slope constant **Λ**, and sigmoid constant *a* are optimized. The optimization is performed using MATLAB (Mathworks, USA), and the optimization algorithm is the interior point methods. The optimized gains are summarized in Table 1.

**Table 1:** Optimal gains for PD-SMC.

Mode	Horizontal Mode			Vertical Mode		
Motion	*x*	*y*	yaw	*z*	roll	pitch
**K**_*P*_	44.46	150.35	11.40	40.39	5.16	43.43
**K**_*D*_	439.86	996.01	60.85	103.06	3.14	41.11
**P**	41.63	40.94	7.91	58.45	2.78	3.62
**Λ**	0.95	0.95	0.94	0.44	0.47	0.30
*a*	15.49	13.28	1.95	1.87	5.49	3.04

### Hovering performance for transient time

To compare the performances of the controllers, the hovering motion was simulated with various tilting durations. The goal of simulation is to maintain a desired position and orientation of the robot starting from an initial position and orientation. The initial position and orientation is *η*_*o*_ = [0.3 m, -0.3 m, 0 m, 30°, -30°, 30°]^T^. The desired position and orientation is *η*_*d*_ = [0 m, 0 m, 0.3 m, 0°, 0°, 0°]^T^. All of the dimensionless errors start from ‘3’, and the convergence of each controller was analyzed. The applied tilting durations are 300, 600, 900, 1200, and 1500 ms, respectively. The control gains of the switching PD controller are chosen from [5].

**Fig 5:**
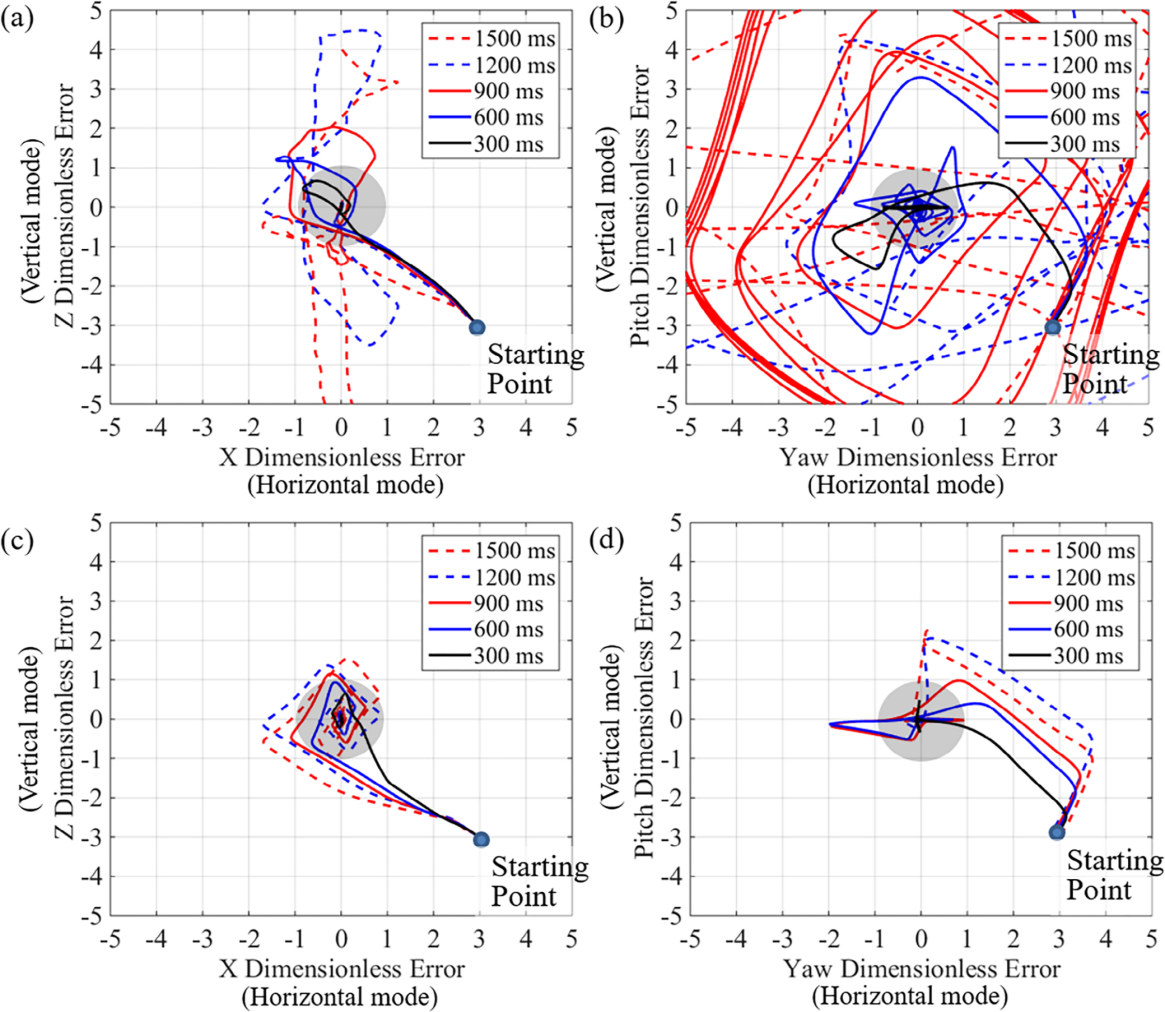
Dimensionless error state with switching for tilting duration. (a) position error and (b) orientation error of the switching PD control, and (c) position error ad (d) orientation error of the switching PD-SMC. Initial position and orientation is the starting point, and each plot shows the degree of convergence to the desired position and orientation with the switching control under various tilting duration.

The dimensionless errors are plotted in Fig 5. The position and orientation error of the switching PD control is shown in Fig 5(A) and 5(B), and the switching PD-SMC results are shown in Fig 5(C) and 5(D), respectively. The horizontal axis denotes the dimensionless error of the x position and yaw angle, which can be controlled in horizontal mode, and the vertical axis denotes the dimensionless error of the z-position and pitch angle, which can be controlled in horizontal mode. These plots show whether the robot converges to the desired position and orientation from the starting point while the thrusters are tilted and controlled via switching control. The grey circle represents the allowable error, which is less than the dimensionless error of 1. The switching PD control cannot maintain stable hovering motion when the tilting duration is slower than 900 ms, as shown in Fig 5(A) and 5(B). On the contrary, the switching PD-SMC shows robust control performance and converges to the desired point even when the tilting duration is 1500 ms, as shown in Fig 5(C) and 5(D). With a tilting duration of 300 ms, the performance of switching PD-SMC is much better than that of switching PD control. Adding the sliding mode term to the PD control has a dramatically positive effect on the hovering performance.

## Experiments

Extensive experiments were performed in a water tank with TTURT in order to verify the simulation results. The position and orientation of TTURT are estimated by a short base line (SBL) system, depth sensor, and inertia measurement unit (IMU). The measured sensor values are given in the earth-fixed frame. The robot is controlled by an NI cRIO-9082 real-time controller (National Instruments, USA).

**Fig 6:**
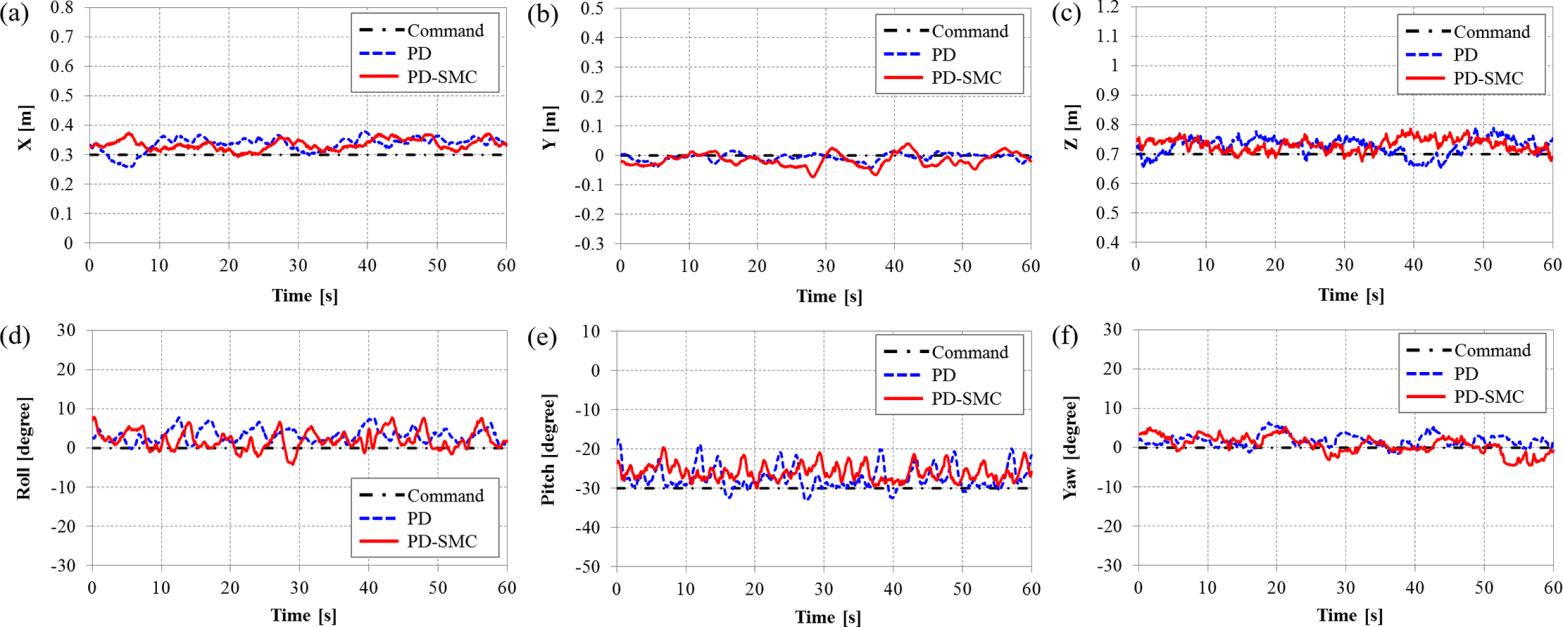
The position and orientation of TTURT with the switching PD control and the switching PD-SMC for a tilting duration of 300 ms. (a) x-position, (b) y-position, (c) z-position, (d) roll angle, (e) pitch angle and (f) yaw angle.

**Fig 7:**
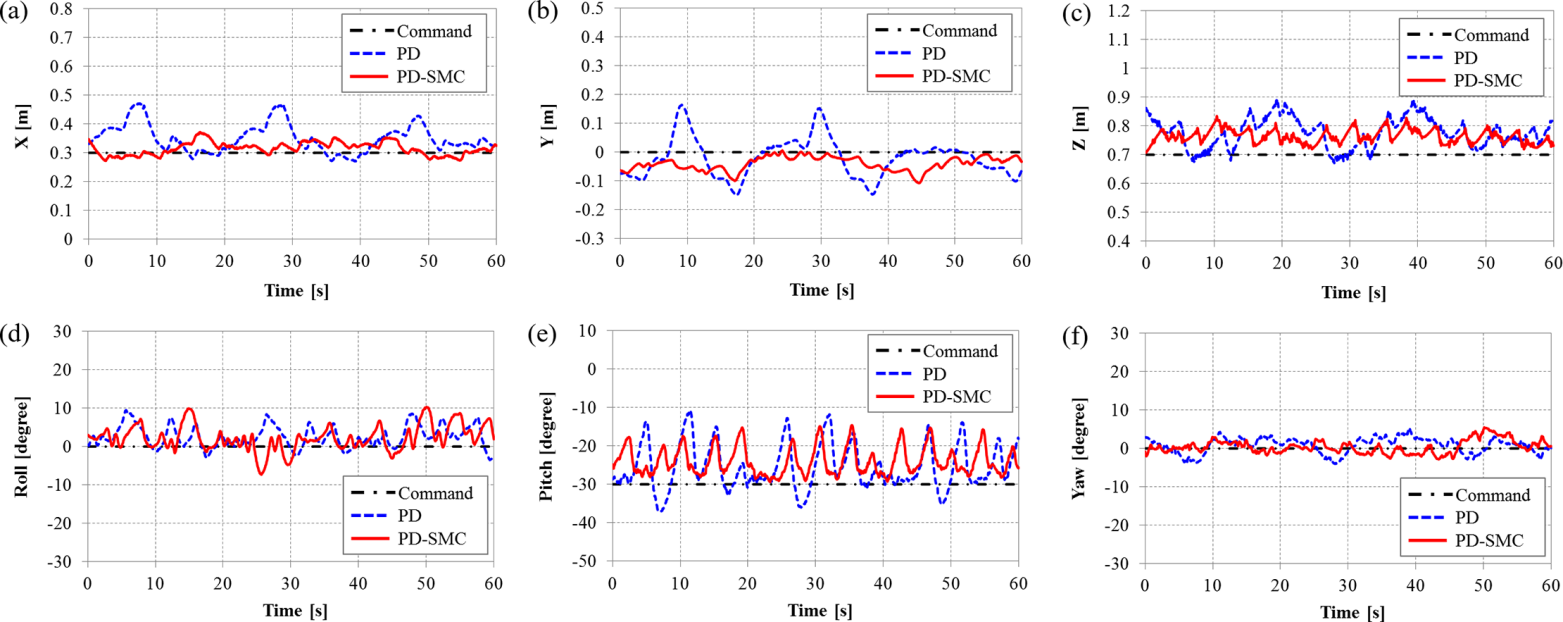
The position and orientation of TTURT with the switching PD control and the switching PD-SMC for a tilting duration of 900 ms. (a) x-position, (b) y-position, (c) z-position, (d) roll angle, (e) pitch angle and (f) yaw angle.

**Fig 8:**
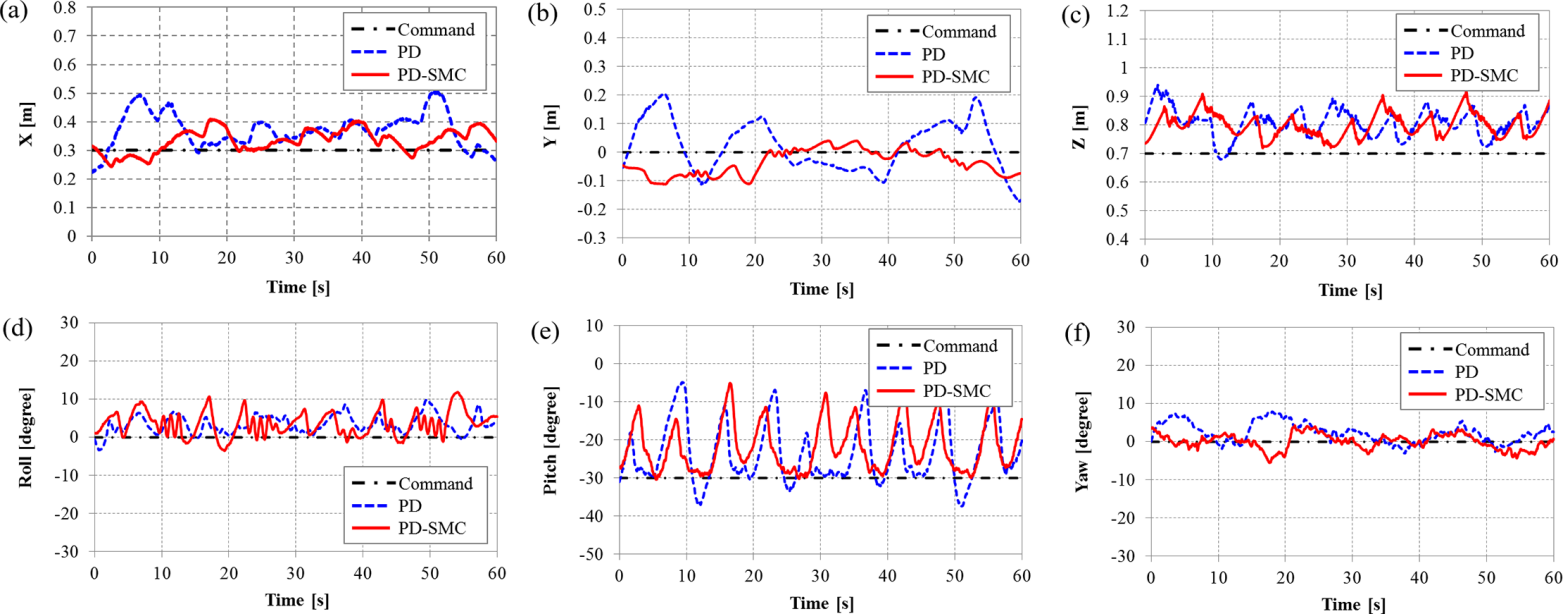
The position and orientation of TTURT with the switching PD control and the switching PD-SMC for a tilting duration of 1500 ms. (a) x-position, (b) y-position, (c) z-position, (d) roll angle, (e) pitch angle and (f) yaw angle.

The goal is to maintain hovering motion with a desired position and orientation, *η*_*d*_ = [0.3 m, 0 m, 0.7 m, 0°, -30°, 0°]^T^. Note that the robot must maintain its motion at a certain pitch angle and hovering motion with this posture is often required to perform underwater tasks. The modeling error for the center of mass acts as a disturbance such as a restoring force [23]. The applied tilting durations are same as in the simulations. The position and orientation with the switching PD and PD-SMC are shown in Figs 6, 7, and 8, where the tilting durations are 300, 900, and 1500 ms, respectively.

**Fig 9:**
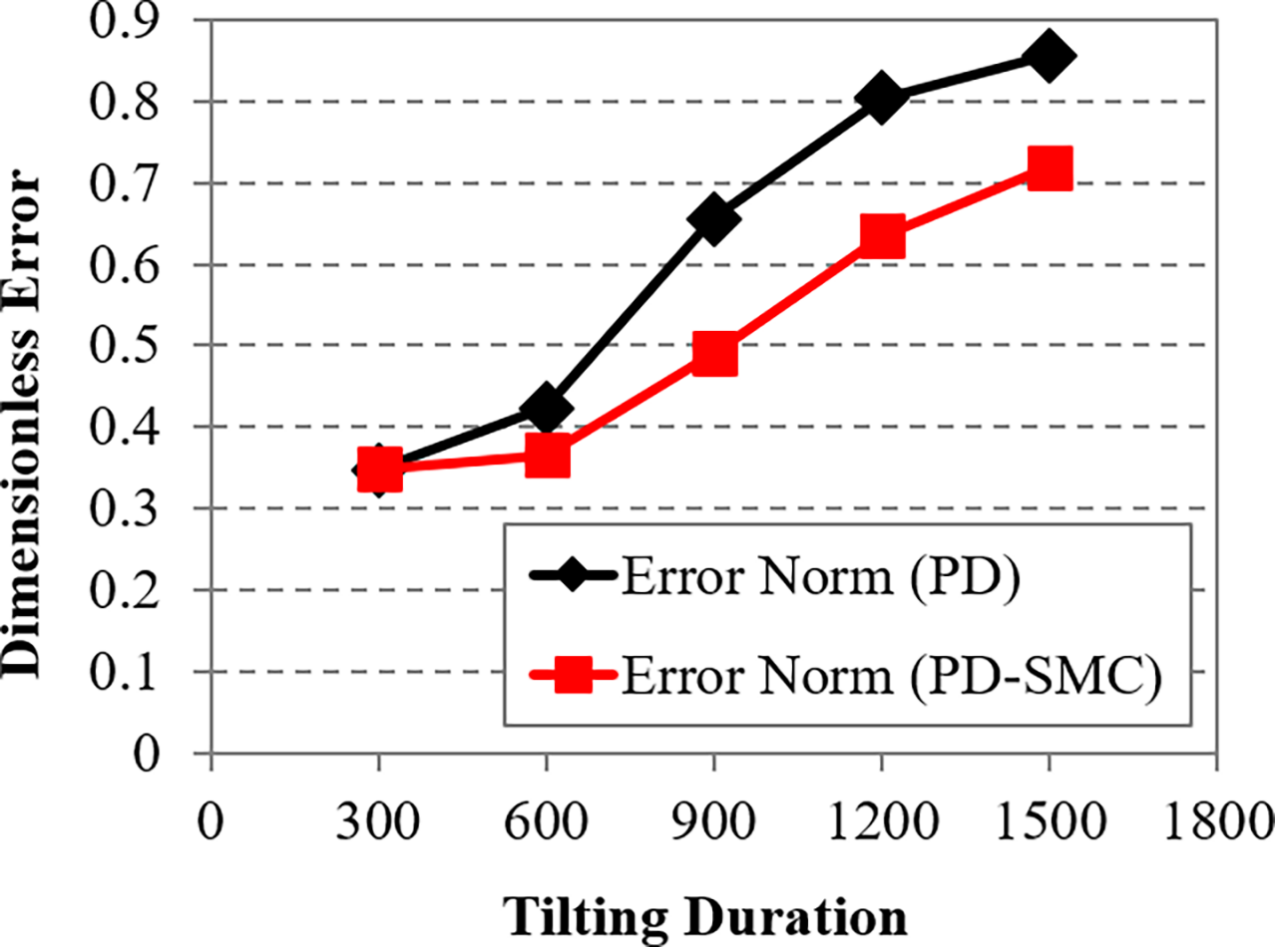
Dimensionless error norm with the switching PD control and the switching PD-SMC for different tilting durations.

When increasing the tilting duration, the oscillating amplitudes of the pitching motions are increased for both controllers. The significant discrepancies in the position control is observed. The switching PD control cannot maintain the position when tilting duration is slower than 900 ms. However, the switching PD-SMC is still relatively stable even with tilting duration of 1500 ms. The norm of dimensionless error from the experiments are shown in Fig 9. Position errors are normalized by reference distance of 0.1 m, and orientation errors are normalized by reference angle of 10°. With the switching PD control, both errors increase sharply with tilting duration slower than 600 ms. Errors of the switching PD-SMC also increase in proportion with the tilting duration but slowly in comparison with the switching PD control. PD-SMC reduced error norm by 25% relative to PD control. The comparison of control performance with the switching PD control and the switching PD-SMC for various tilting duration can be seen from a video clip as shown in S1 File. The experimental data verify the robustness of the proposed switching PD-SMC against various tilting durations.

## Conclusions

This paper proposed a switching PD-SMC method with a sigmoid function and applied the proposed controller to an underwater robotic platform with tilting thrusters. Since The tilting thruster mechanism has limited tilting speed, the control performance is considerably influenced by the tilting speed. The model-free PD-SMC was designed to ensure robust control performance in combination with parameter optimization. The sigmoid function in the sliding mode was introduced to reduce chattering. The stability of each PD-SMC subsystem was theoretically validated via Lyapunov stability theory. Extensive simulations and experiments demonstrated that the switching PD-SMC was more robust despite variation of the tilting speed, while the switching PD control failed with tilting duration of 900 ms or more. When the tilting duration becomes larger, the performance gap between two methods becomes significantly increased.

## Appendix

The matrices in the motion equations of TTURT can be derived based on rigid body dynamics and hydrodynamics. Many terms in the motion equations are simplified using the mentioned assumptions such as neutral buoyancy, coincidence of center of mass and buoyancy, and symmetric shape of three planes. The added mass and inertia terms are derived via strip theory. The hydrodynamic damping is analyzed by simulation with computational fluid dynamics software, ANSYS CFX (ANSYS Inc., USA). The matrices are as follows [4]:

$$J=\left[\begin{array}{cccccc} c\psi c\theta & -s\psi c\phi +c\psi s\theta s\phi & s\psi s\phi +c\psi c\phi s\theta & 0 & 0 & 0\\ s\psi c\theta & c\psi c\phi +s\phi s\theta s\psi & -c\psi s\phi +s\theta s\psi c\phi & 0 & 0 & 0\\ -s\theta & c\theta s\phi & c\theta c\phi & 0 & 0 & 0\\ 0 & 0 & 0 & 1 & s\phi t\theta & c\phi t\theta \\ 0 & 0 & 0 & 0 & c\phi & -s\phi \\ 0 & 0 & 0 & 0 & s\phi /c\theta & c\phi /c\theta \end{array}\right],$$ (A.1)

$$M=\left[\begin{array}{cccccc} 96.3 & 0 & 0 & 0 & 0 & 0\\ 0 & 133.04 & 0 & 0 & 0 & 0\\ 0 & 0 & 168.57 & 0 & 0 & 0\\ 0 & 0 & 0 & 4.47 & 0 & 0\\ 0 & 0 & 0 & 0 & 9.26 & 0\\ 0 & 0 & 0 & 0 & 0 & 8.1 \end{array}\right],$$ (A.2)

$$C(v)=\left[\begin{array}{cccccc} 0 & 0 & 0 & 0 & 168.57w & -133.04v\\ 0 & 0 & 0 & -168.57w & 0 & 96.3u\\ 0 & 0 & 0 & 133.04v & -96.3u & 0\\ 0 & 168.57w & -133.04v & 0 & 8.1r & -9.26q\\ -168.57w & 0 & 96.3u & -8.1r & 0 & 4.47p\\ 133.04v & -96.3u & 0 & 9.26q & -4.47p & 0 \end{array}\right],$$ (A.3)

$$D(v)=\left[\begin{array}{cccccc} 34.55\left|u\right| & 0 & 0 & 0 & 0 & 0\\ 0 & 104.4\left|v\right| & 0 & 0 & 0 & 0\\ 0 & 0 & 146.5\left|w\right| & 0 & 0 & 0\\ 0 & 0 & 0 & 0.68\left|p\right| & 0 & 0\\ 0 & 0 & 0 & 0 & 5.34\left|q\right| & 0\\ 0 & 0 & 0 & 0 & 0 & 3.07\left|r\right| \end{array}\right],$$ (A.4)

where, s, c, and t in (A.1) mean sine, cosine, and tangent function, respectively.

## Supporting information

S1 File Comparison of control performance with the switching PD control and the switching PD-SMC for various tilting duration. (MP4)
